# Supplementary material for: PAX7, a Key for Myogenesis Modulation in Muscular Dystrophies through Multiple Signaling Pathways: A Systematic Review
Source: Int J Mol Sci. 2023 Aug 22;24(17):13051. doi: 10.3390/ijms241713051 (PMC10487808; doi:10.3390/ijms241713051)
Supplement: Supplementary file 1 [file ijms-24-13051-s001.zip › ijms-2478678-supplementary.pdf]

Systematic Review

PAX7, A Key for Myogenesis Modulation in Muscular Dystrophies through Multiple Signaling Pathways: A Systematic Review

Nor Idayu A. Rahman[1], Lam Chung Liang[1], Nadiah Sulaiman[1], Nur Atiqah Haizum Abdullah[1], Fazlina Nordin[1], Shahrul Hisham Zainal Ariffin[2] and Muhammad Dain Yazid[1]*

[1]Centre for Tissue Engineering & Regenerative Medicine, Faculty of Medicine, Universiti Kebangsaan Malaysia Medical Centre, Jalan Yaacob Latif, 56000 Cheras, Kuala Lumpur, Malaysia.
[2]Centre of Biotechnology & Functional Food, Faculty of Science and Technology, Universiti Kebangsaan Malaysia, 43600 Bangi, Selangor, Malaysia.
* Correspondence: Tel.: +603 9145 6995; Fax: +603 9145 7678; Email: dain@ukm.edu.my

Supplementary Material

Exclusion Criteria

The exclusion criteria are applied in order, in which pulled articles are subjected to these criteria. Any article that did not meet any of the exclusion criteria is therefore included in the synthesis.

Exclusion Criteria 1
Exclude if the article is published before 2013.

Exclusion Criteria 2
Exclude one of the articles if the title is a duplicate. In cases where all three databases generate the exact article, only include one.

Exclusion Criteria 3
Exclude if the article is not written in English.

Exclusion Criteria 4
Exclude if the article is not an original article.

Exclusion Criteria 5
Exclude if the article is a published review, book chapter, reports, editorials, method and protocols, and conference abstract.

Exclusion Criteria 6
Exclude if the focus of the review is not about:
1. Pax7 gene and/or protein expression.
2. Signaling pathway affecting or affected by Pax7 regulation.

Exclusion Criteria 7
Exclude if the approach of the study of the article does not include *in vivo* and *in vitro* studies.

Exclusion Criteria 8

Exclude if the outcome of the study is irrelevant. Exclusion Criteria 7 is applied after full-text reading of the articles by two independent reviewers prior to article selection.
